# Influence of gastric emptying on the control of postprandial glycemia: physiology and therapeutic implications

**DOI:** 10.1590/S1679-45082014RB2862

**Published:** 2014

**Authors:** Marcos Antonio Tambascia, Domingos Augusto Cherino Malerbi, Freddy Goldberg Eliaschewitz

**Affiliations:** 1 Universidade Estadual de Campinas, Campinas, SP, Brazil; 2 Hospital Israelita Albert Einstein, São Paulo, SP, Brazil; 3 Centro de Pesquisas Clínicas, São Paulo, SP, Brazil

**Keywords:** *Diabetes mellitus*/therapy, Hyperglycemia, Gastric emptying, Glucagon-like peptide 1

## Abstract

The maintenance of glucose homeostasis is complex and involves, besides the secretion and action of insulin and glucagon, a hormonal and neural mechanism, regulating the rate of gastric emptying. This mechanism depends on extrinsic and intrinsic factors. Glucagon-like peptide-1 secretion regulates the speed of gastric emptying, contributing to the control of postprandial glycemia. The pharmacodynamic characteristics of various agents of this class can explain the effects more relevant in fasting or postprandial glucose, and can thus guide the individualized treatment, according to the clinical and pathophysiological features of each patient.

## RESUMO

A manutenção da homeostase glicêmica é complexa e envolve, além da secreção e da ação da insulina e do glucagon, mecanismos hormonais e neurais, que regulam a taxa de esvaziamento gástrico. Esse mecanismo depende de fatores extrínsecos e intrínsecos. A secreção do peptídeo 1 semelhante ao glucagon regula a velocidade de esvaziamento gástrico, de modo a contribuir para o controle da glicemia pós-prandial. As características farmacodinâmicas dos diversos agentes dessa classe podem explicar os efeitos mais relevantes na glicemia de jejum ou pós-prandial e, portanto, podem orientar o tratamento individualizado, de acordo com as características clínicas e fisiopatológicas de cada paciente.

## INTRODUCTION

Blood glucose control involves a complex mechanism, encompassing not only the secretion and action of insulin and glucagon,^(1)^ but also regulation of the gastric emptying rate.^(2)^

Since 1915,^(3)^ the variability of blood glucose after a dose of oral glucose is known to depend on changes in the gastric emptying rate, data confirmed by several subsequent studies.^(4,5)^

Incretin-based treatment (both with dipeptidil-peptidase-4 inhibitors –DPP4i, and glucagon-like peptide-1 - GLP1) involves the secretion of insulin by a glucose-dependent mechanism. This action, along with the reduction in the levels of glucagon,^(6)^ gives these drugs a promising role in the treatment of type 2 *diabetes mellitus*. Besides primarily metabolic actions, this treatment affects other systems and, therefore, has aroused increasing interest. One of these actions is the reduction in gastric emptying rate.^(7)^

Recently, the American Diabetes Association and the European Association for the Study of Diabetes^(8)^ have emphasized the need to customize the treatment approach for diabetes, basing treatment targets and medication on the pathophysiologic aspects peculiar to each case. Reviewing these foundations is, therefore, an essential task.

### Regulation of gastric emptying

The gastric emptying rate is variable and depends on the content of the meal. Thus, if the fluid ingested is rich in glucose or calories, emptying is delayed. This inhibition determines a constant rate of energy absorption, of approximately 2 to 3kcal/minute; glucose infusion in the duodenum inhibits gastric emptying proportionally to the amount infused. This sensitive mechanism depends on extrinsic and intrinsic factors.

The extrinsic path, also known as “ileal brake mechanism”,^(9)^ depends on neural and hormonal feedback induced by the interaction of nutrients in the lumen of the small intestine, and is calorie-dependent. When food reaches the bowel, L and K cells of the distal small intestine produce GLP-1 and glucose-dependent insulinotropic peptide - GIP, which act on several tissues. In the hypothalamus, the peptides reduce appetite and send cholinergic and peptidergic signs to the vagus, inhibiting antral motility and stimulating pyloric motility. These actions contribute to the inhibition of gastric emptying.^(10)^ GLP1 and GIP stimulate insulin secretion and inhibit glucagon secretion. Moreover, they stimulate secretion of an amyloid pancreatic peptide, amylin.^(11)^ Insulin has a satiety effect on the central nervous system, while amylin decreases gastric emptying, due to vagal action.^(12)^

Intrinsic mechanisms involve effects of hyperglycemia or hypoglycemia. Hyperglycemia stimulates secretion of insulin and amylin, reducing the secretion of glucagon; it also decreases the secretion of ghrelin, which reduces gastric emptying, via parasympathetic signal. Physiologically, ghrelin increases the gastric emptying rate.^(13)^ Thus, in addition to the calorie aspect (extrinsic), blood glucose variations (intrinsic) may, through hypothalamic actions, increase or decrease appetite and activate the parasympathetic system that controls gastric emptying. This delicate neural and hormonal interaction prevents postprandial hyperglycemia in normal individuals.

### Regulation of gastric emptying in diabetes

Some studies showed that pronounced hyperglycemia (>250mg/dL) affects motility of the esophagus, stomach, small intestine, colon and gallbladder, both in normal^(14)^ and type 1^(15)^ and 2 diabetic individuals.^(16)^ It was suggested initially that hyperinsulinemia also delays gastric emptying,^(17)^ but recent studies in type 1 diabetic individuals^(18,19)^ have challenged these observations.

In healthy individuals, the behavior of blood glucose and incretin levels is highly dependent on the exposure of the small intestine to carbohydrates.^(20)^ Hence, higher overloads lead to more intense elevations in the levels of GLP1, resulting in progressive reduction in gastric emptying rate, a physiologic mechanism that contributes to blood glucose homeostasis. These data were reproduced in individuals with well-controlled type 2 diabetes,^(21)^ who received intraduodenal infusions of increasing amounts of glucose. This important study demonstrated that concentrations of insulin, GLP1 and GIP increased with intraduodenal overload of glucose, both in normal and diabetic individuals.

Preservation of this control mechanism in patients with diabetes opens possibilities of treatment with strategies that influence the rate of duodenal exposure to carbohydrates.

### Prospects in the treatment of postprandial hyperglycemia in diabetes

The DECODE study^(22)^ made it clear that postprandial hyperglycemia is an independent risk factor for macrovascular disease, and its control is essential for reducing cardiovascular mortality in diabetic individuals. The approaches for controlling postprandial glucose levels include prandial insulin analogues,^(23)^ acarbose – of which action occurs, in part, due to increased levels of GLP1,^(24)^ pramlintide^(25)^ – of vagal action and, more recently, treatments based on the incretin system, especially GLP1.

The mechanism of action of GLP1 during fasting essentially involves the secretion of glucose-dependent insulin and inhibition of glucagon. Recent studies have demonstrated, however, that its action in the postprandial state occurs through deceleration of gastric emptying, leading to reduced entry of glucose in circulation.^(26)^ The concomitant use of prokinetic agents, such as metoclopramide, domperidone, cisapride and erythromycin, blocks the effect of GLP1 to control postprandial blood glucose,^(27)^ demonstrating the importance of its influence in gastric emptying.

Continuous exposure to high concentrations of GLP1 leads to a pronounced loss of ability to decrease gastric emptying.^(28)^ This is due to induction of tachyphylaxis at the vagus level, and causes an attenuation of the response after chronic administration.

The pharmacokinetic and pharmacodynamic analyses of GLP1 demonstrate that this class of drugs is comprised of molecules with variable binding profiles to receptor of GLP1, which probably explains some differences of action among them. The prolonged action with the receptor leads to desensitization of the inhibitory gastric effect, but neither due to the anorectic effect that occurs through hypothalamic GLP1 receptors^(29)^, nor to the desensitization of the effect on insulin secretion and fasting blood glucose control. On the other hand, molecules, whose interaction with the receptor is short have greater effect on the reduction of gastric emptying – and, consequently on the postprandial glucose control – because they do not lead to desensitization.^(28)^

The comparison between agents with faster (lixisenatide) *versus* more prolonged (liraglutide) action^(30)^ shows differences in their impact on the regulation of fasting and postprandial blood glucose levels, and may guide individualized treatment, according to the clinical and pathophysiological characteristics of each patient.

## CONCLUSION

We are heading toward treatment of diabetes based on individualization of medications. The choice of the best pharmaceutical will be based on understanding the pathophysiology of each case and the mechanism of action of medications. GLP1 analogues with prolonged action may benefit patients with high fasting glucose levels and who need to lose weight. Fast action analogues will be a very good option to correct cases of postprandial hyperglycemia.
